# Development of Loop-Mediated Isothermal Amplification (LAMP) Assay for Rapid and Sensitive Identification of Ostrich Meat

**DOI:** 10.1371/journal.pone.0100717

**Published:** 2014-06-25

**Authors:** Amir Abdulmawjood, Nils Grabowski, Svenja Fohler, Sophie Kittler, Helga Nagengast, Guenter Klein

**Affiliations:** Institute of Food Quality and Food Safety, University of Veterinary Medicine, Hannover, Hannover, Germany; Ehime University, Japan

## Abstract

Animal species identification is one of the primary duties of official food control. Since ostrich meat is difficult to be differentiated macroscopically from beef, therefore new analytical methods are needed. To enforce labeling regulations for the authentication of ostrich meat, it might be of importance to develop and evaluate a rapid and reliable assay. In the present study, a loop-mediated isothermal amplification (LAMP) assay based on the *cytochrome b* gene of the mitochondrial DNA of the species *Struthio camelus* was developed. The LAMP assay was used in combination with a real-time fluorometer. The developed system allowed the detection of 0.01% ostrich meat products. In parallel, a direct swab method without nucleic acid extraction using the HYPLEX LPTV buffer was also evaluated. This rapid processing method allowed detection of ostrich meat without major incubation steps. In summary, the LAMP assay had excellent sensitivity and specificity for detecting ostrich meat and could provide a sampling-to-result identification-time of 15 to 20 minutes.

## Introduction

In Europe, ostrich meat has been consumed since the times of the Roman Empire. However, farming (for meat and leather) started in the 1860s. Nowadays, ostriches are farmed inside and outside Africa, and ostrich meat has become a popular foodstuff, mostly because of its low fat and low cholesterol content and as an alternative during the worldwide bovine spongiforme enzephalopathie (BSE) crisis during the 1990s.

In 1995, the Food and Agriculture Organization of the United Nations (FAO) claimed that ostrich farming was at a comparable level to turkey farming in the 1920s and predicted a fair economic development. At present there are no official data on the present status of ostrich farms worldwide. According to the World Ostrich Organisation (http://world-ostrich.org/demand.htm), the annual production of ostrich meat amounts to 12,000 to 15,000 metric tonnes, with South Africa being the main source (60%) for this meat type. Recently, outbreaks of avian epidemic in traditional ostrich-farming countries (e.g. South Africa) have lead to trade bans with important trade partners, e.g. the EU. This has, on the one hand, lead to shifts in the international ostrich meat trade. On the other hand, ostrich farmers in temperate zones have benefited from this development, although European production is still far from being self-sufficient. Thus, currently, Germany, where ostrich meat is relatively popular due to its nutritional properties, relies on both imported and local productions.

The four subspecies of *Struthio camelus* are grouped into red-necked (*S.c. camelus* and *S.c. massaicus*) and blue-necked (*S.c. molybdophanes* and *S.c. australis*) ostriches. The typical farm ostrich, also called “African Black” and termed *S.c. domesticus*, is the result of hybridizing *S.c. camelus* with *S.c. australis*, and is considered to be of the blue-neck type [1].

While the composition of ostrich meat is relatively similar to poultry, the sensory properties resemble beef, and, when a given lot consists of dressed cuts (which is the rule rather than the exception), a clear distinction between the two meat types is difficult for the meat inspectors. This difficulty may lead to two problems: first, ostrich meat imported from Algeria, Burkina Faso, the Cameroons, the Central African Republic, Chad, Mali, Mauritania, Morocco, Niger, Nigeria, Senegal and Sudan could include wild specimens of *S.c. camelus* which is protected by CITES and, within the EU, via Commission Reg. (EU) No 750/2013 of July 29, 2013. Second, since ostrich meat is more expensive than that of traditional domestic species, there is the chance of fraud. In fact, a recent study [2] in an attempt to authentify the species claimed in South African meat products sold locally that only eight of n = 23 ostrich samples actually contained this species. The most popular substitutes were beef (n = 9), local antelope meat (n = 3) and kangaroo meat (n = 2).

Thus, efficient methods to identify ostrich meat without doubt are necessary. One way to accomplish this is by using mitochondrial DNA (mtDNA) analysis. According to Carney and others [3], Rigaa and others [4], Yoshizaki and others [5] and Abdulmawjood and Buelte [6], mtDNA analysis requires the isolation of the mtDNA molecule and a digestion of the mtDNA with a variety of restriction endonucleases. The resulting fragment patterns are then examined for polymorphisms within and among examined populations.

A comparable combination of PCR amplification of mtDNA and RFLP analysis has been used successfully in animal species identification studies (Wilson and others [7]; Ram and others [8]; Yoshizaki and others [5]; Carrera and others [9]–[11]; Cespedes and others [12]; Abdulmawjood and Buelte [6]). Abdulmawjood and others [13] used a quantitative real-time PCR for gender differentiation in pig meat and meat products.

The present study was designed to develop and validate a loop-mediated, isothermal amplification (LAMP) method for identifying ostrich meat. For this design a conserved mitochondrial DNA region of the *cytochrome b* gene was taken as a base. This region had already been used successfully for identification and phylogenetic studies for many different organisms [2].

## Materials and Methods

### Investigated samples

The total DNA was isolated from 27 fresh and frozen ostrich meat samples obtained from supermarkets and via internet in Germany. This included fresh roast meat (n = 2), fresh fillet steak (n = 2) (Straussenhof, Kotzenbach, Germany), frozen roast meat (n = 8) (Vitaline-GmbH, Teutschenthal, Germany), frozen goulash (n = 12) (Schneider Fine Food GmbH, South Africa), and ostrich steak (n = 3) (Krause Meat International Food, Lüneburg, Germany). In addition, four reference samples of African Black Ostrich (n = 2) and Blue-neck Ostrich (n = 2) were used. These reference samples were kindly supplied by Dr. Y. Hemberger, Ostrich Production (Namibia) (PTY) Ltd., Namibia. In addition, eight samples were heat-treated, fried with cooking oil and spices (n = 2), or boiled at 100°C for 30 min (n = 2), 60 min (n = 2) and 120 min (n = 2), respectively. For the exclusivity test, cow, pig, sheep, goat, turkey, chicken, dog, cat, horse and deer reference DNAs were used. These reference DNA of the non-ostrich species were obtained from the institute's DNA collection.

### Positive and negative predictive values (PPV and NPV)

The PPV was calculated as (number of true positives)/(number of true positives + number of false positives) ×100, and the NPV was calculated as (number of true negatives)/(number of true negatives + number of false negatives) ×100. The accuracy was calculated as (number of true positives + number of true negatives)/(total number of patients) ×100.

### DNA extraction and template preparation

The DNA was isolated using the DNeasy tissue Isolation Kit (Qiagen, Darmstadt, Germany) according to the manufacturer's instructions. Briefly, 25 mg of the meat sample was lyzed and followed by binding of the DNA to the column. After two washing steps the DNA was eluted with 100 µl elution buffer. The DNA quantity was measured using Qubi Flourometer (Invitrogen, Darmstadt, Germany).

### Design of LAMP assay oligonucleotide primers

The development oligonucleotide primers for the LAMP assay was based on the reference sequence of ostrich *cytochrome b* gene subunit published in the National Center for Biotechnology (NCBI) GenBank under accession no. AF069431.1. The sequence alignments were performed with the *cytochrome b* gene of different meat animal species including sequences of cow (*Bos taurus*; KC153977.1), pig (*Sus scrofa scrofa*; AY534296.1), sheep (*Ovis aries*; X56284.1), turkey (*Meleagris gallopavo*; L08381.1), chicken (*Gallus gallus*; L08376.1), dog (*Canis lupus familiaris*; JF342899.1), cat (*Felis catus*; X82296.1), horse (*Equus ferus caballus*, KC202959.1), buffalo (*Bubalus bubalis* FJ467729.1) and lama (*Lama glama*; Y19184.19).

A set of six oligonucleotide primers, including two outer primers (forward primer F3-Ost and backward primer B3-Ost), two inner primers (forward inner primer FIP-Ost and backward inner primer BIP-Ost), and two loop primers (forward loop primer LoopF-Ost and backward loop primer LoopB-Ost), were designed using LAMP Designer software, ver. 1.10 (PREMIER Biosoft, CA, USA). These primers were selected to be specific for ostrich *cytochrome b* gene (Table 1). The oligonucleotide primers were synthesized by Eurofins MWG Operon (Ebersberg, Germany). The oligonucleotide sequences were submitted to the NCBI GenBank for specificity testing, including broad range and comparative genome Basic Local Alignment Search Tool (BLAST) analysis.

### LAMP reaction and amplification condition

LAMP reaction was carried out with a total volume of 25 µl of the reaction mixture containing 0.5 µl each of F3-Ost and B3-Ost primer (25 pmol/µl) equivalent to 5 µM final concentration, 2.0 µl each of FIP-Ost and BIP-Ost primer (25 pmol/µl) equivalent to 20 µM final concentration, 1.0 µl each of LoopF-Ost and LoopB-Ost primer (25 pmol/µl) equivalent to 10 µM final concentration. 15 µl Isothermal Master Mix Iso-001 (Optigene, UK). 3 µl DNA was added as a template. The LAMP assay was run at 65°C for 30 min with a melting curve analysis step (annealing curve 98°–80°C ramping at 0.1 per min) in a portable real-time fluorometer (Genie II, Optigene, UK).

### Analytic sensitivity of the ostrich LAMP assay

LAMP reaction of a serially diluted ostrich-DNA were performed using the same conditions mentioned above. A serial dilutions (10^−1^–10^−5^) were prepared using TE buffer (pH 8.0). The amount of the DNA was estimated by using QubiTM Flourometer.

### Internal reference material (IRM) and limit of detection (LOD) for ostrich meat

To determine the detection limit, different internal reference materials (IRM) were prepared. Meat mixtures of 100 g of bovine meat were prepared with 10%, 5%, 1%, 0.1% and 0.01% of ostrich meat, respectively. The mixtures were homogenized using Grindomix (Retsch, Haan, Germany). The DNA was isolated in triplicate from each concentration using the DNeasy tissue Isolation Kit and investigated in two runs with the LAMP conditions mentioned above.

### Swab and HYPLEX LPTV buffer test method

As a rapid and simple processing test, sterile cotton swabs were used directly on an ostrich steak piece and on minced bovine meat containing 5% ostrich meat. Briefly, the dry swab was removed from the package and rubbed and rolled firmly several times over the surfaces of the meat sample. The swab was then placed into a 500 µl HYPLEX LPTV buffer tube (AmplexDiagnostics, Gars, Germany), and broken or cut, leaving the swab head inside the tube. The tube was shaken several times by hand (without vortexing). 3 µl was applied as a template directly, after 5 and after 10 min in the LAMP reaction.

## Results

### Specificity and the analytical sensitivity of the LAMP assay

**Figure 1:**
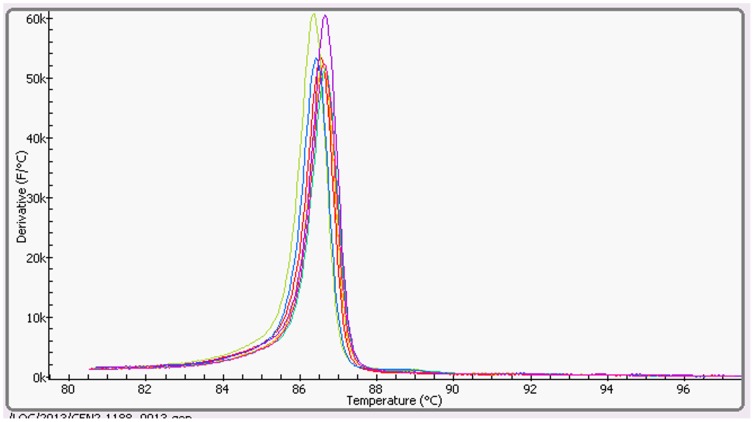
The anneal curve reactions of different ostrich meat reference DNA. The products showed a melting temperature of 86.5°C (±0.25).

Using the oligonucleotide primer set designed for ostrich meat, all 31 ostrich meat DNA samples were identified correctly positive. No amplification of any of the 10 non-ostrich meat samples was observed. By melting curve analysis which termd by Genie II anneal curve analysis, no significant differences among the different ostrich samples were obtained. The melting temperature was 86.5°C (±0.25°C) for the specific ostrich amplicon (Figure 1).

The amplification rate and the analytical sensitivity of the LAMP assay were calculated on the basis of a dilution row, the amount of the DNA ranging from 35 ng/µl (10^0^) to 0.35 pg/µl (10^−5^) for ostrich meat DNA. The analytic sensitivity of the LAMP assay for ostrich DNA was 0.35 pg/reaction. The detection probability was determined to be 100%. This concentration could be detected by a detection time of approx. 16∶42 (±1.17) min (Table 2).

**Table 1:** Oligonucleotide primers sequences used for LAMP assay.

Designation	Sequence	Primer long	Melting temp
F3-Ost	5′-CCT CCC ATC TCC CTC AAA-′3	18 bp	56.0°C
B3-Ost	5′-TAA GAG CCA TAG TAG AGT CCT C-′3	22 bp	58.4°C
FIP-Ost	5′-GGC GAC GGA TGA GAA TGC TTT CTA ACA GGG CTC CTA CTA G-′3	40 bp	74.6°C
BIP-Ost	5′-ACA CAT GCC GGA ACG TAC AGT ACA GAT GAA GAA GAA GGA TGC-′3	42 bp	73.3°C
LoopF-Ost	5′-GTG TAG TGT CGG CTG TGT AA-′3	20 bp	57.3°C
LoopB-Ost	5′-GAT TTA TCC GCA ATC TCC ATG C-′3	22 bp	58.4°C

**Table 2:** Analytical sensitivity of the LAMP assay using serial dilution of ostrich DNA.

Dilution step	DNA amount pg/µl	Detection time (mm:ss)				
		run1	run 2	run3	mean	Sd ±
10^−1^	3500	7∶38	7∶37	7∶8	7∶52	0.20
10^−2^	350	9∶08	8∶53	9∶2	8∶94	0.29
10^−3^	35	10∶23	10∶53	11∶01	10∶59	0.32
10^−4^	3.5	11∶38	13∶08	13∶1	12∶52	0.81
10^−5^	0.35	15∶53	18∶08	15∶66	16∶42	1.17

All eight heat treated ostrich samples were positive by LAMP assay, the detection time ranging between 08∶43 min and 10∶58 min. The mean of the detection time of the heat treated samples was 09∶37 (±0.42) min.

### Limit of Detection of the LAMP assay

**Figure 2:**
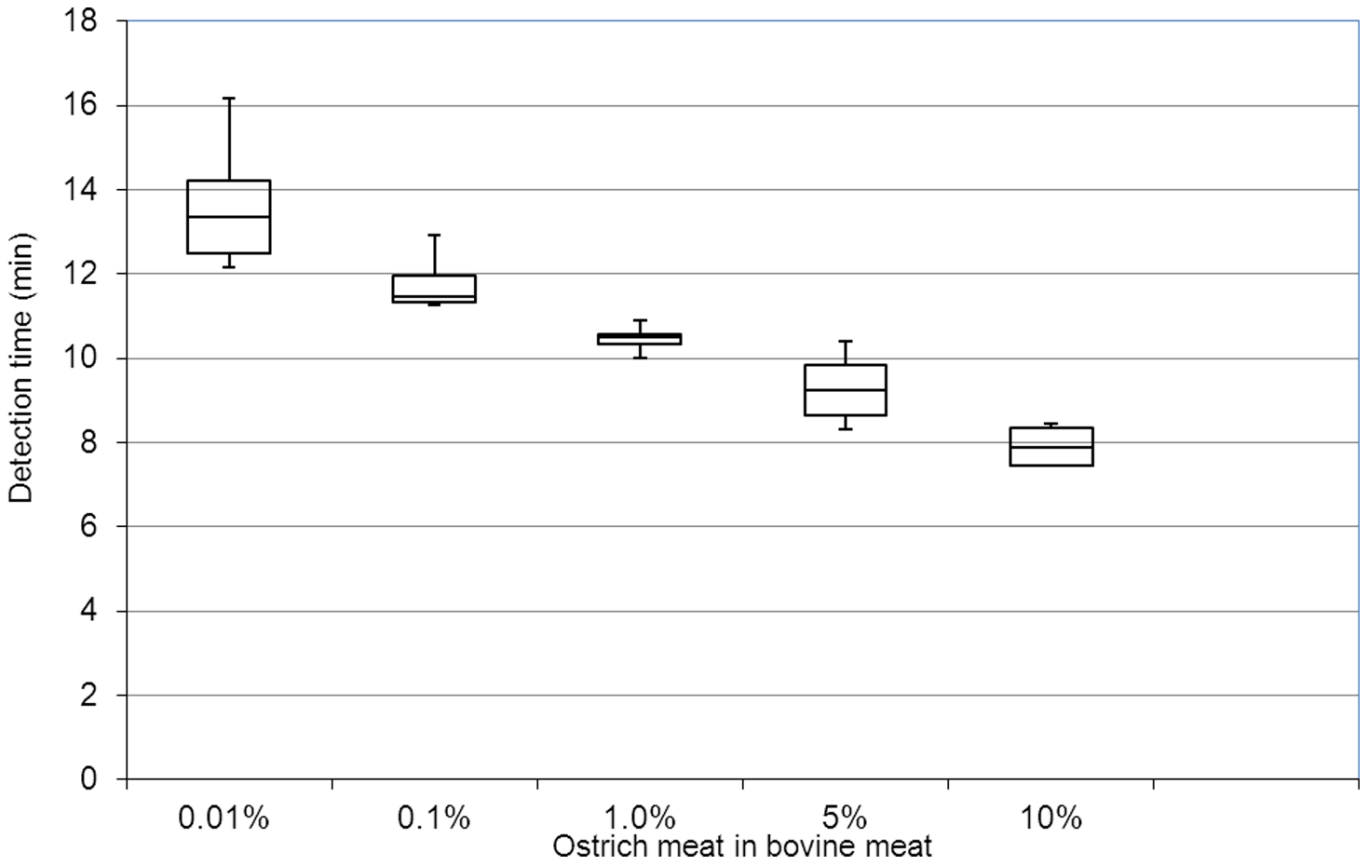
Box plots of detection time of LAMP assay with a detection limit up to 0.01% of ostrich. The Box plots was generated by using six replecates for each concentration.

To estimate the limit of detection (LOD) of the developed ostrich LAMP assay, bovine meat samples with varying additives of ostrich meat (10%, 5%, 1%, 0.1% and 0.01%) were investigated (six replicates for each concentration). The time of detection of the varying ostrich meat concentrations ranged from 13.36 (±1.7) min and 7.87 (±0.89) min for sample containing 0.01% and 10% ostrich meat, respectively (Figure 2). The LAMP assay allowed the detection of ostrich meat at a concentration of 0.01%, yielding a detection probability of 100% (all six LAMP reactions were positive). With the LAMP assay the negative control as well as the meat without added ostrich meat yielded no fluorogenic signal.

### Swab and HYPLEX LPTV buffer test method

**Figure 3:**
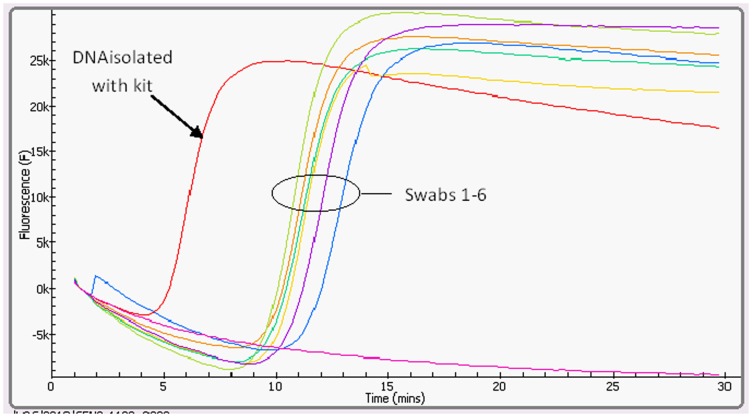
Amplification signal of ostrich meat DNA using the direct swab and HYPLEX buffer test method. The red curve is the signal of ostrich DNA isolated with DNeasy tissue Isolation Kit (detection time of 6∶18 min). The other positive curves in the oval shap are the direct swab samples with a detection time of 11∶03–12∶18 min. The pink curve is the negative control (see table 3).

For a rapid and simple sample preparation, the direct application of dry swab method yielded a clear fluorogenic signal with a detection time of 11∶26 min when the DNA was applied directly, 11∶18 min after 5 min of incubation at room temperature and 12∶40 after 10 min of incubation. While the DNA isolated by DNeasy tissue Isolation Kit showed a detection time of 6∶18 min, all reactions displayed an annealing temperature of 86.73°C (±0.04). The swab samples of the minced bovine meat containing 5% ostrich meat were also positive, showing a detection time of 11∶52 min (±0.07 min) (Table 3 and Figure 3).

**Table 3:** Time of detection and annealing temperatures of a dry swab and HYPLEX LPTV buffer test method.

Sample	Incubation time in HYPLEX buffer	Detection time (mm:ss)	Annealing temperature
Swab 1	Direct without incubation	11∶18	86.71°C
Swab 2	Direct without incubation	11∶33	86.76°C
Swab 3	5 min at room temperature	11∶03	86.76°C
Swab 4	5 min at room temperature	11∶33	86.76°C
Swab 5	10 min at room temperature	13∶03	86.66°C
Swab 6	10 min at room temperature	12∶18	86.76°C
Kit DNA*	-	6∶18	86.71°C

* Ostrich DNA isolated with DNeasy tissue Isolation Kit.

## Discussion

An accurate method for identifying meat species is of great importance in forensic science cases. Food legislation in Germany and other EU countries establishes that meat products should be labeled correctly using official nomenclature. Regulation (EC) 178/2002, regarding food traceability, requires that the origin of all raw materials may be identified readily at all stages of the meat processing chain.

Among recently available molecular detection methods, LAMP is one of the most widely researched ones and has been well-characterized, offering significant support during the development process. The publication of diagnostic assays in different fields using LAMP increased from one publication in the year 2000 to 210 in 2013, according to the PubMed, National Center for Biotechnology Information NCBI (as of Jan. 2014). This method has appeared to be an alternative to the PCR-based methods, not only in food safety testing but also in other application fields of diagnostical assays.

To our knowledge, there are no published reports on the use of the LAMP assay for the species identification of meat products. Up to now species identification in meat samples has been based on PCR-RFLP analysis, species-specific conventional PCR or Real-time PCR [6], [14], [15]. In their analysis, D'Amato and others [2] used a PCR and sequencing method which was based on *cytochrome b* region, together with the *cytochrome c* oxidase subunit I (*COI*) region. However, this method is expensive and labor-intensive.

In the present study, all ostrich meat samples obtained from retailers as well as the reference ostrich DNA were positive using the newly designed LAMP assay. None of the investigated DNA of other animal species for control purposes showed any cross reaction with the developed assay. The calculated PPV and NPV were 100% with the investigated samples. The developed assay identified the species *Struthio camelus* in general with a consideration of interspecies variation. The high specificity of LAMP assay for the authentification of ostrich meat is due to the fact that a set of six primers with eight binding sites must hybridize correctly to their target gene sequence before DNA amplification occurs. The analytical sensitivity of this assay was <1 pg DNA. This is due to the six primers used in this study instead of four employed in many other reports. The use of six primers produces more amplicons during amplification reaction. However, this high reproduction of the target amplicon in turn bears an elevated risk of cross contamination of subsequent samples by aerosolized products (i.e. false-positive results) [16]. In order to avoid this, the present study used a closed system tube (Genie II) in which reaction vessels were not opened either during the analysis or after the reaction run. Another benefit of the real-time fluorometer system in comparison to conventional gel electrophoresis analysis is the semi-quantitative results of the analysis of different meat products. LOD was up to 0.01%, i.e. 1 g of ostrich meat may be detected in 10 kg of meat product.

The LAMP reaction was also carried out with DNA extracted from heat-treated ostrich meat. A strong signal appeared even after heat treatment of the meat prior to DNA extraction at 100°C up to 120 min. In addition, DNA was also extracted successfully from fried meat with oil and spices. This demonstrates the robustness of LAMP in the presence of PCR inhibitor substances like salt, spices and cooking oil. Tartaglia and others [17] and Abdulmawjood et al. [15] could also amplify the dog and cat meat DNA from heat-treated samples. The rapid amplification time and its low susceptibility to inhibition factors (when compared to classical PCR [18], [19]) are noteworthy properties for the application suitability of this assay in the investigation of food samples in restaurants or even of canned meat samples.

The swab and HYPLEX LPTV buffer test method has been evaluated with two types of meat, steak and minced bovine meat containing 5% ostrich meat. The results of the present study show that different times of incubation of the swabs in HYPLEX LPTV buffer did not have a significant effect on the detection time. This rapid lysis procedure worked well with both minced meat and steak, giving positive results by LAMP without the need for extraction. However, the amplification times were longer than those using extracted DNA with a kit. Nonetheless, the fluorescent signals of all samples were positive after approx. 12 min. In spite of the slightly longer amplification times used with the swab and HYPLEX LPTV buffer processing method (when compared with extracted DNA), this approach still provided an accurate identification result within 20 min using portable equipment. The uses of Genie II in food analysis will allow the food controllers to carry out the test in the field, e.g. in restaurants or retail shops. In addition to the advantages mentioned by Njiru et al. [20] which also included lower costs for instruments the LAMP technology may be combined readily with a real-time fluorometer like Genie II.

## Conclusion

The use of LAMP amplification assays of a conserved region of the *cytochrome b* gene provides a simple, quick and reliable direct identification of ostrich specimens. This method could be used to identify inappropriately-labeled ostrich meat directly at the restaurant or at retail level. This is even possible with cooked, fried and spiced samples.
